# A rare PBX1 variant identified in adulthood: a case report

**DOI:** 10.3389/fmed.2025.1604376

**Published:** 2025-11-03

**Authors:** Ah-Rim Han, Youngyoon Moon, Yang-Gyun Kim, Sang-Ho Lee, Ju-Young Moon, Sung Jig Lim, Yu Ho Lee, Su Woong Jung

**Affiliations:** ^1^Department of Medicine, Graduate School, Kyung Hee University, Seoul, Republic of Korea; ^2^Division of Nephrology, Department of Internal Medicine, Kyung Hee University Hospital at Gangdong, Seoul, Republic of Korea; ^3^Division of Nephrology, Department of Internal Medicine, College of Medicine, Kyung Hee University, Seoul, Republic of Korea; ^4^Department of Pathology, College of Medicine, Kyung Hee University, Seoul, Republic of Korea; ^5^Department of Internal Medicine, CHA Bundang Medical Center, CHA University, Seongnam, Republic of Korea

**Keywords:** PBX1, rare variant, early-onset chronic kidney disease, proteinuria, focal segmentalglomerulosclerosis, tubular necrosis

## Abstract

Abnormalities in *PBX1* represent a monogenic cause of congenital anomalies of the kidney and urinary tract (CAKUT). However, their phenotypic heterogeneity poses a challenge for timely detection, particularly in the absence of overt anomalies in the kidney and urinary tract. Here, we present a 28-year-old male diagnosed with a rare *PBX1* nonsense variant identified during the evaluation of early-onset chronic kidney disease. As part of the initial workup for decreased renal function and proteinuria, a kidney biopsy was performed, revealing focal segmental glomerulosclerosis (FSGS) and acute tubular necrosis without an identifiable cause. He was initially treated with renin-angiotensin system inhibitors, followed by glucocorticoid and/or cyclosporine therapy for four years. Despite these interventions, his serum creatinine levels gradually increased without any improvement in proteinuria. Genetic testing, performed seven years after the initial visit, revealed a rare *de novo* heterozygous *PBX1* variant, p.Arg93Ter (c.277C > T), classified as likely pathogenic. Reverse phenotyping identified cryptorchidism and dysmorphic external ears, both of which are extrarenal manifestations commonly associated with *PBX1*-related CAKUT. Although this variant is predicted to be deleterious, it is flagged as escaping nonsense-mediated decay, which may explain the absence of apparent structural anomalies in the kidneys. PBX1 is prominently expressed in interstitial and endothelial cells in both fetal and adult human kidneys, and its function is not directly implicated in podocyte or tubular cell biology. Therefore, the inadvertent pathological findings in this genetic disorder may be attributed to reduced nephron endowment and/or disturbance in reciprocal cellular interactions. This case broadens the phenotypic spectrum of *PBX1*-related disorders and highlights its renal manifestations, further expanding the clinical heterogeneity of FSGS.

## 1 Introduction

PBX1 encodes the Pre-B-cell leukemia transcription factor 1, a master regulator involved in diverse developmental and pathological processes (1). The genetic defects in PBX1 are implicated in a broad spectrum of renal and extrarenal manifestations, including varying degrees of renal hypoplasia or dysplasia, and anomalies affecting the genitourinary, branchial, and skeletal systems, collectively referred to as congenital anomalies of the kidney and urinary tract (CAKUT). However, the clinical presentation of *PBX1*-related disease can be subtle and may lack overt structural abnormalities of the kidney or urinary tract, posing diagnostic challenges in clinical practice.

Here, we report the case of a 28-year-old man who was initially diagnosed with focal segmental glomerulosclerosis (FSGS) and acute tubular necrosis of unknown etiology, and was later found to carry a *de novo PBX1* nonsense variant. This case provides histopathologic insight into the renal features associated with a *PBX1* variant and highlights the importance of genetic evaluation in adults presenting with early-onset chronic kidney disease (CKD) of undetermined cause.

## 2 Case presentation

**Figure 1:**
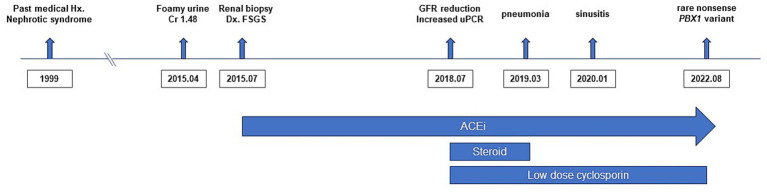
Timeline of the patient’s clinical course. The timeline summarizes the major clinical events, diagnostic evaluations, and treatments.

**Figure 2:**
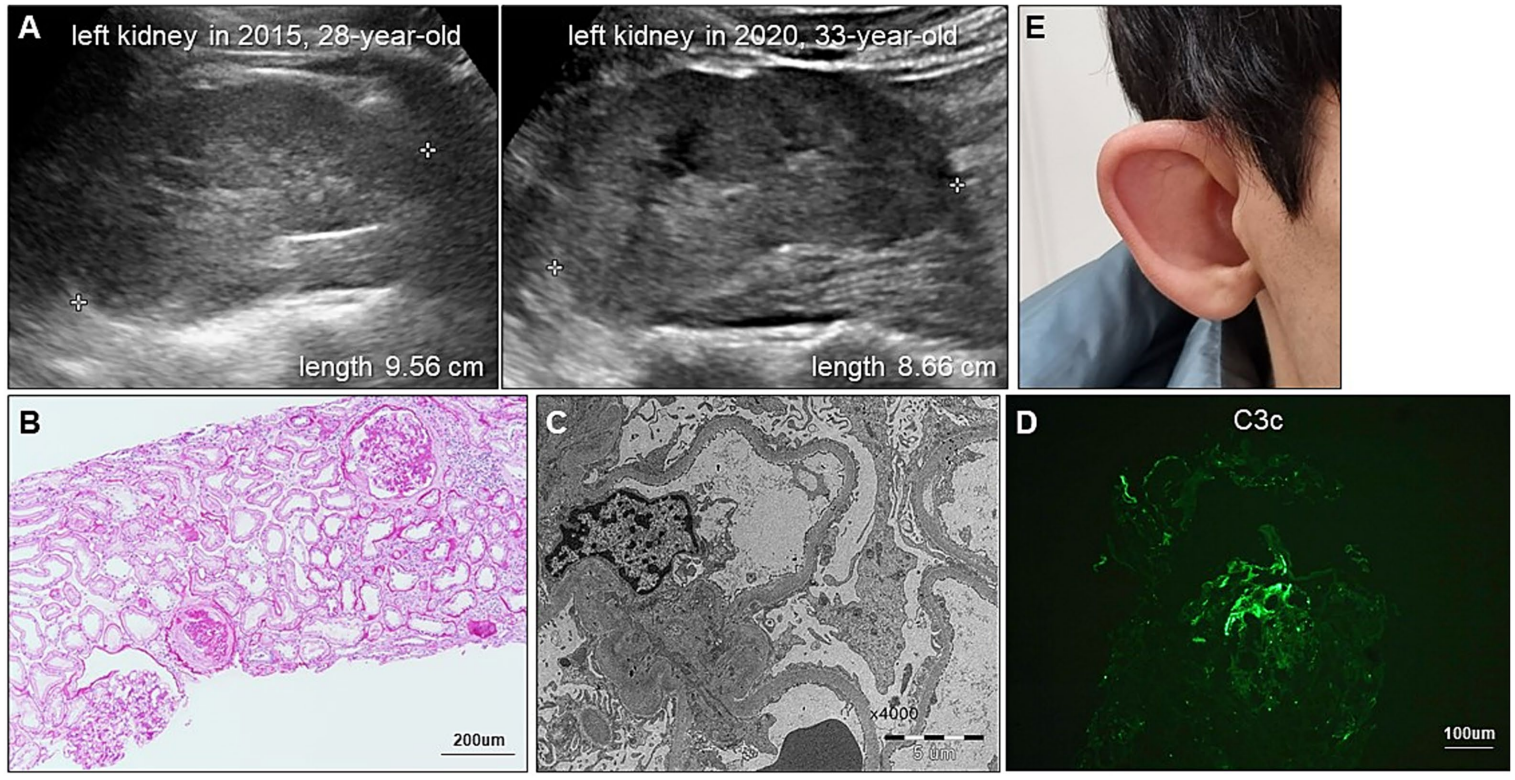
Renal and extrarenal manifestations of CAKUT in our patient. **(A)** Serial renal ultrasonography demonstrates progressive reduction in kidney size. **(B)** Periodic acid-Schiff staining shows global and segmental glomerulosclerosis and tubular injury with immune cell infiltration. **(C)** Electron micrograph shows diffuse foot process effacement without electron-dense deposits. **(D)** Immunofluorescence staining for C3 shows segmental granular deposits, regarded as nonspecific trapping in sclerotic areas. **(E)** Photograph displays a dysmorphic external ear with an underdeveloped auricle, consistent with microtia.

A 28-year-old man presented to our outpatient clinic with foamy urine in 2015 (Figure 1). His medical history included an unexplained episode of nephrotic syndrome at the age of 12, after which he had been well until this visit. There was no family history of kidney disease, and he did not exhibit signs of edema or obesity. His vital signs were stable, with a blood pressure of 127/72 mmHg (normal range, <120/80 mmHg). Laboratory evaluations revealed an elevated serum creatinine level of 1.52 mg/dL (reference range, 0.7–1.2 mg/dL), corresponding to an estimated glomerular filtration rate of 54.9 mL⋅min^−1^ ⋅1.73 m^−2^ calculated by the CKD Epidemiology Collaboration equation, along with subnephrotic range proteinuria of 844 mg/gCr (reference range, <150 mg/gCr) on spot urine analysis. Serological tests were unremarkable, except for a positive antinuclear antibody titer of 1:80. Ultrasonography revealed increased cortical echogenicity in relatively small kidneys, measuring approximately 9.5 cm in length (reference range, 10**–**13 cm) (Figure 2A).

To determine the etiology of CKD, a kidney biopsy was performed, revealing FSGS accompanied by diffuse foot process effacement and a moderate degree of tubular necrosis (Figures 2B,C). Immunofluorescence staining showed segmental granular C3c deposition (F0201, Dako), while staining for immunoglobulin G (IgG) (F0315, Dako), IgA (F0204, Dako), IgM (F0203, Dako), C1q (F0254, Dako), and C4c (F0169, Dako) was negative. In the absence of electron-dense deposits on electron microscopy, the C3c staining was interpreted as nonspecific trapping in areas of sclerosis (Figure 2D). Over a three-year follow-up period, the patient was treated with renin-angiotensin system inhibitors and lipid-lowering agents; however, his proteinuria slightly increased up to 1,135 mg/gCr. For the unknown etiology of FSGS, a 6-month course of prednisolone (starting from 30 mg once daily and tapered out) in combination with cyclosporin 50 mg to 75 mg twice daily was empirically administered in 2018. Thereafter, cyclosporin monotherapy 50 mg to 75 mg twice daily was continued. Unfortunately, this pharmacological intervention did not yield significant improvement in his proteinuria, and rather his serum creatinine levels gradually escalated to 1.98 mg/dL. In parallel with the decline in renal function, follow-up ultrasonography in 2020 showed further reduction in kidney size, measuring approximately 8.6 cm (Figure 2A).

**Figure 3:**
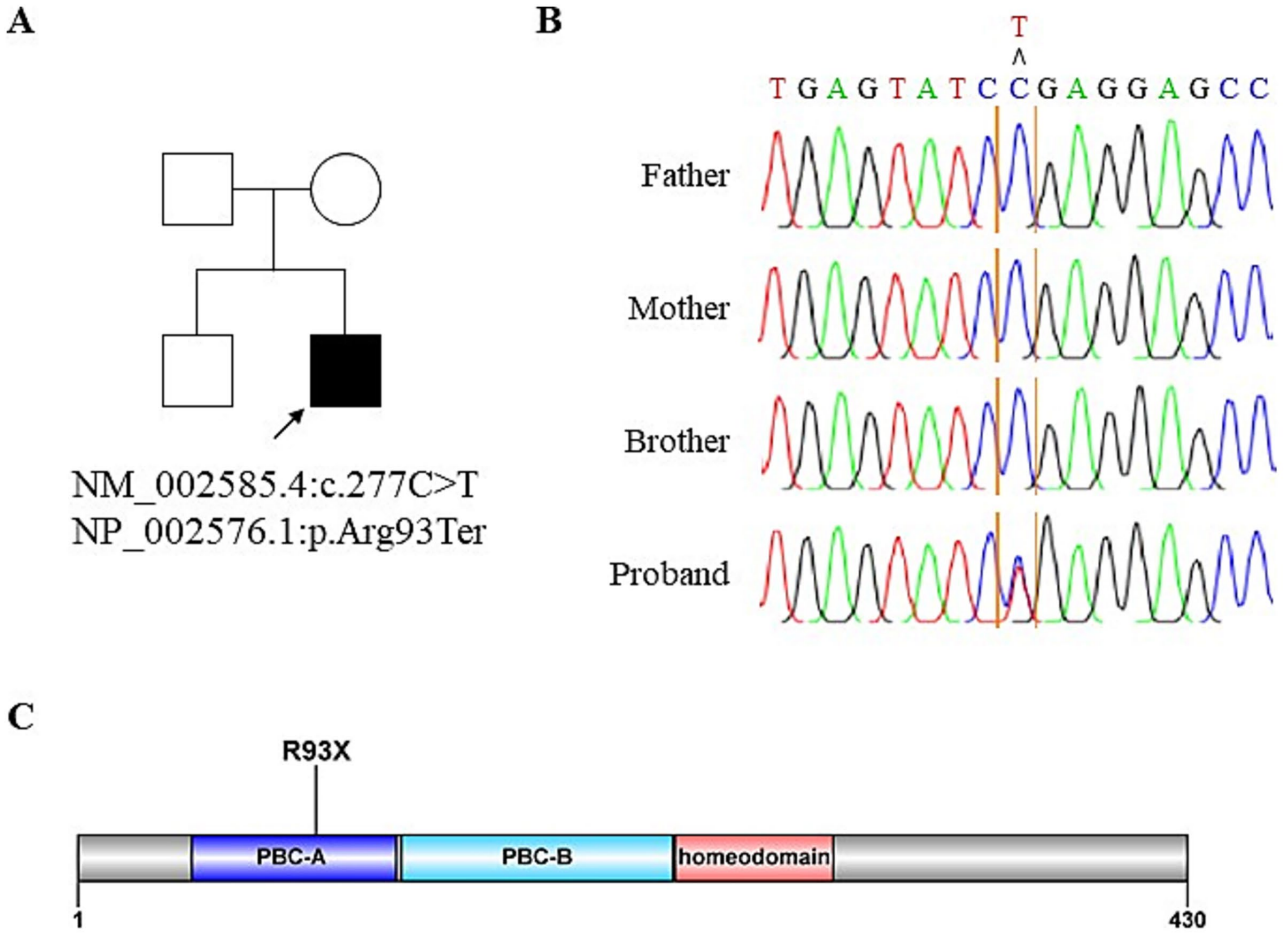
Identification of novel *PBX1* variant. **(A)** Pedigree of the affected individual. **(B)** Sanger sequencing confirms *de novo PBX1* variant in the patient. Blood samples from the patient and his family are used for Sanger sequencing. **(C)** Schematic of the PBX1 protein with a *PBX1* variant in the proband.

In 2022, whole exome sequencing was performed using the 3B-EXOME (3billion Inc.) on DNA extracted from a buccal swab sample, which was provided free of charge by a pharmaceutical company. The analysis identified a rare heterozygous nonsense variant in *PBX1*, located at chromosome 1:164,761,742 (GRCh37), resulting in a p.Arg93Ter substitution (NM_002585.4:c.277C > T) (Supplementary Table 1). Sanger sequencing of blood samples from the patient and his family confirmed the presence of this variant and revealed that his variant is sporadic (Figures 3A,B). The variant was predicted to be deleterious according to the Combined Annotation Dependent Deletion version 1.6 Phred score of 36. Additional history taking from his family informed that the patient had cryptorchidism at birth and had been teased for dysmorphic external ears (Figure 2E) during adolescence, both of which are extrarenal phenotypes of CAKUT. Examination revealed an underdeveloped auricle, a feature of microtia, although the patient did not complain of hearing impairment.

These results prompted the discontinuation of futile cyclosporin use. Throughout the four-year use of immunosuppressants, the patient frequently experienced sore throat and cough, and occasionally required antibiotics for pneumonia and acute sinusitis. The follow-up serum creatinine levels remained constantly around 2.0 mg/dL with the persistence of a similar degree of proteinuria, ranging from 600 mg/gCr to 800 mg/gCr until the last visit in 2025.

## 3 Discussion

This case is a representative example demonstrating that early-onset chronic kidney disease with no apparent cause may be attributed to a genetic defect. As illustrated by this patient, the diagnosis of *PBX1*-related disease can be challenging, even though associated extrarenal anomalies were present and a renal biopsy was performed. Until molecular diagnosis was made, subtle dysmorphisms were overlooked, a detailed medical history was not obtained, and unnecessary immunosuppressants were prescribed. This case highlights the importance of incorporating genetic testing into the diagnostic evaluation for early-onset CKD or FSGS without apparent causes.

The variant identified in our case was a novel variant that was not listed in gnomAD v2.1.1. *PBX1* is a well-established monogenic cause of CAKUT, with the majority of *PBX1*-related cases, including ours, arising *de novo* (2–8). PBX1 haploinsufficiency, caused either by microdeletion encompassing the entire gene or by splice-site, frameshift, and nonsense variants that lead to premature termination of protein synthesis, results in structural renal abnormalities, most commonly manifested as bilateral renal hypoplasia, dysplasia, ectopia, or horseshoe kidneys (2–6).

Although information on the presence of renal hypoplasia at birth was unavailable, the renal phenotype in our patient remained largely subclinical until the onset of renal function decline and the development of proteinuria, despite the presence of a truncating *PBX1* variant. The relatively mild phenotype observed in this case may be explained by two potential mechanisms. First, although this variant occurs early in the coding sequence (Figure 3C), Ensembl has annotated it as a nonsense-mediated decay escaping variant, suggesting the potential production of a truncated protein with residual function. Second, given the sporadic occurrence of this variant, somatic mosaicism cannot be excluded, as reported in another case involving a *PBX1* nonsense variant (7). Similarly, a different *PBX1* nonsense variant, p.Arg288Ter (c.862C > T), was identified in a 36-year-old Chinese woman during evaluation for mildly reduced renal function (8). In that case, no apparent abnormalities of the kidney and urinary tract were observed except renal malrotation, demonstrating the phenotypic variability in the severity of *PBX1*-related disease.

PBX1 is a transcription factor that is implicated in kidney development. Immunohistochemistry image from the Human Protein Atlas (http://www.proteinatlas.org) showed its nuclear expression in glomerular and interstitial cells (Supplementary Figure 1A). Specifically, single-cell and single-nucleus RNA-sequencing (9, 10) have shown strong *PBX1* expression in the interstitial and endothelial cells in fetal and adult human kidneys, contrasting with its low expression in podocytes and tubular cells (Supplementary Figures 1B,C and Supplementary Table 2). Accordingly, the histopathologic features of FSGS and acute tubular necrosis in a patient with a *PBX1* variant were unexpected. As the kidneys develop through reciprocal interactions between nephrons, ureteric, stromal, and endothelial progenitor cells, we speculated that abnormal cell behavior in stromal or glomerular endothelial cells may disrupt tubular and podocyte development and maintenance. In addition, a reduced number of nephrons at birth may impose a cumulative burden on remaining glomeruli over time, ultimately contributing to the development of secondary FSGS.

FSGS is a histopathologic pattern of injury rather than a specific disease entity. Our case expands the known clinical spectrum of FSGS by illustrating that this histopathologic pattern may emerge in the context of CAKUT-related genetic disorders. The KDIGO 2021 guideline (11) suggests genetic testing in selected FSGS cases, targeting podocyte and glomerular basement membrane genes (12). Our case suggests that, in cases of FSGS without a clear secondary cause, broader kidney-specific gene panels that include CAKUT-associated genes, such as *PBX1*, should be considered to ensure accurate diagnosis and guide appropriate management.

In conclusion, this case demonstrates the diagnostic challenges that nephrologists may face when evaluating early-onset CKD without a clear etiology. Broader implementation of genetic testing in routine clinical practice could help mitigate these challenges, thereby improving patient care.

## Data Availability

The original contributions presented in the study are included in the article/Supplementary material, further inquiries can be directed to the corresponding author/s.
