# Supplementary material for: A rare PBX1 variant identified in adulthood: a case report
Source: Front Med (Lausanne). 2025 Nov 3;12:1604376. doi: 10.3389/fmed.2025.1604376 (PMC12620263; doi:10.3389/fmed.2025.1604376)
Supplement: Supplementary file 1 [file Data_Sheet_1.docx]

Supplementary Material

**
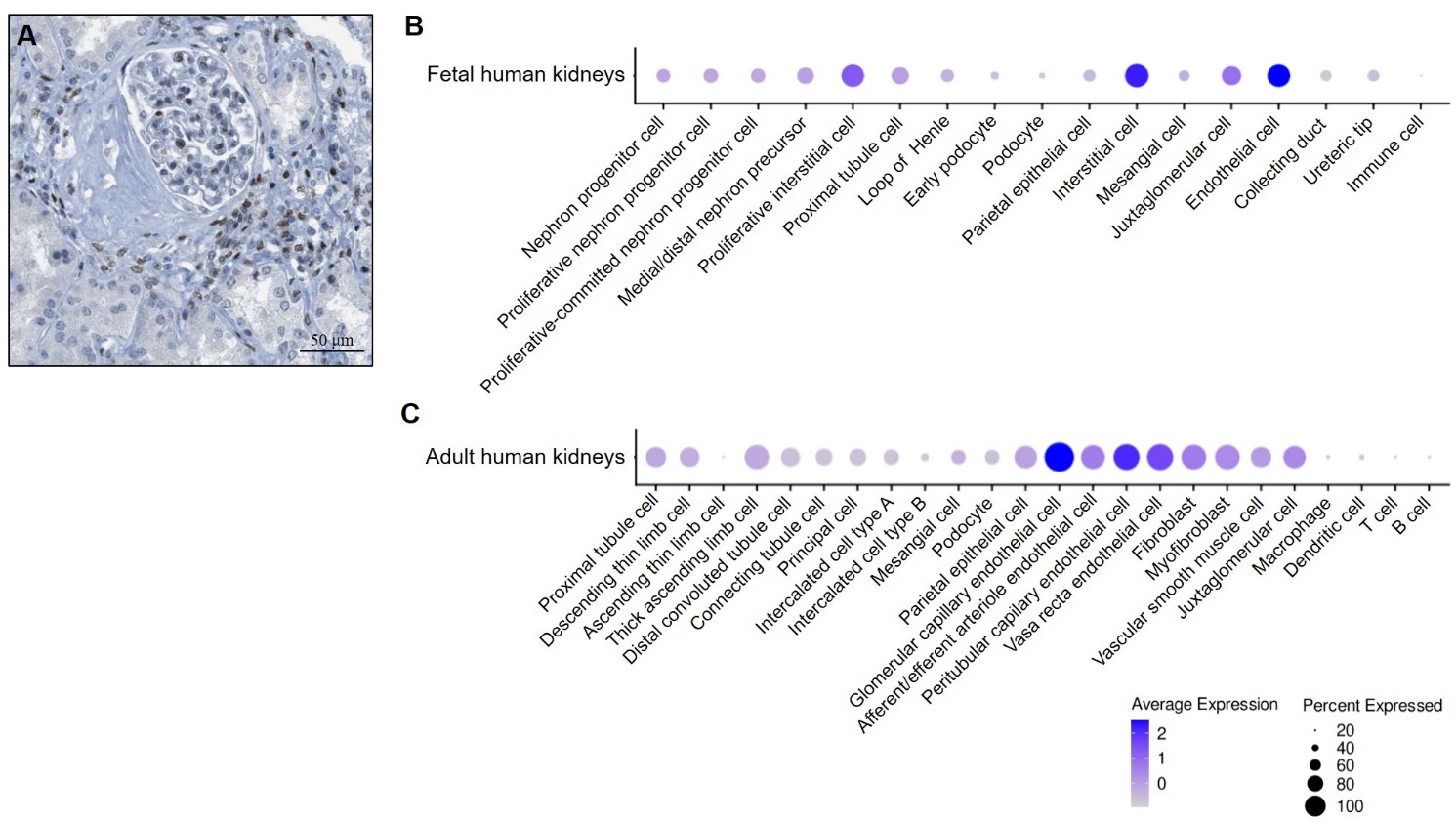
Supplementary Figure** **1. PBX1 expression in human kidneys.**

(A) Protein-level expression of PBX1 in adult human kidneys. The image was obtained from the Human Protein Atlas (<http://www.proteinatlas.org>).
(B,C) Transcriptional expression of *PBX1* in fetal and adult human kidneys. Dot plots display *PBX1* expressions across various cell types. Dot color represents the average expression level, and dot size indicates the proportion of cells expressing *PBX1*. Publicly available single-cell RNA-seq data from 15–17-week fetal human kidneys (1) and single-nucleus RNA-seq data from normal adult kidneys (2) were used for the analysis.


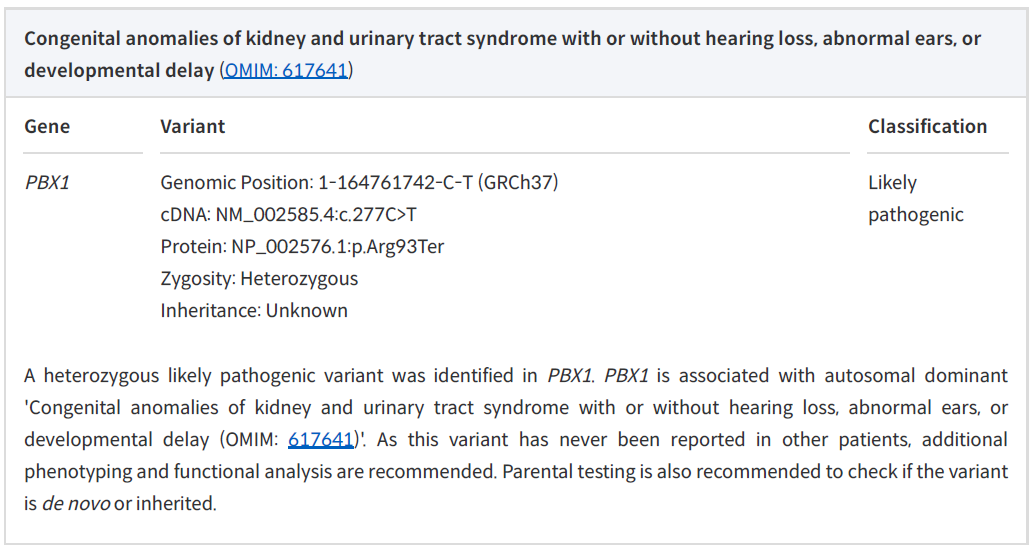
Supplementary Table 1. 3B-EXOME report

Supplementary Table 2. Transcriptional profile of PBX1 in cell types where it is differentially expressed

A. Fetal human kidneys

|  | avg_log_2_FC | pct.1 | pct.2 | p_val_adj |
| --- | --- | --- | --- | --- |
| Proliferative interstitial cells | 0.450 | 0.66 | 0.427 | 1.17592E-21 |
| Interstitial cells | 1.039 | 0.684 | 0.381 | 7.2102E-263 |
| Endothelial cells | 0.961 | 0.658 | 0.417 | 1.65053E-92 |
|  |  |  |  |  |

B. Adult human kidneys

|  | avg_log_2_FC | pct.1 | pct.2 | p_val_adj |
| --- | --- | --- | --- | --- |
| Glomerular capillary endothelial cell | 1.800 | 0.889 | 0.639 | 0 |
| Afferent/efferent arteriole endothelial cell | 0.586 | 0.747 | 0.643 | 5.39626E-14 |
| Peritubular capillary endothelial cell | 1.577 | 0.799 | 0.635 | 0 |
| Vasa recta endothelial cell | 1.335 | 0.805 | 0.633 | 0 |
| Fibroblast | 0.707 | 0.771 | 0.63 | 0 |
| Myofibroblast | 0.367 | 0.768 | 0.641 | 8.1737E-69 |

The table summarizes statistical information on PBX1 expression shown in Figure 4. To identify cell types in which *PBX1* is differentially expressed in fetal and adult human kidneys, we used the FindAllMarkers function implemented in Seurat v4.1.0, using single-cell RNA-seq from 15–17-week fetal kidneys (1) and single-nucleus RNA-seq from adult kidneys (2). For the adult samples, we extracted single-nucleus transcriptomes from the full dataset, excluding adaptive/maladaptive, degenerative, and cycling states of each cell type for analysis. Differential expression analysis was conducted using the Wilcoxon rank sum test, with thresholds set at a minimum difference in detection fraction of 0.1 between one cell type and all others and an adjusted P value < 0.05.

**Reference**

1. Tran T, Lindstrom NO, Ransick A, De Sena Brandine G, Guo Q, Kim AD, et al. In Vivo Developmental Trajectories of Human Podocyte Inform In Vitro Differentiation of Pluripotent Stem Cell-Derived Podocytes. Dev Cell. 2019;50(1):102-16 e6.

2. Lake BB, Menon R, Winfree S, Hu Q, Melo Ferreira R, Kalhor K, et al. An atlas of healthy and injured cell states and niches in the human kidney. Nature. 2023;619(7970):585-94.
